# Effect of adiposity and physical fitness on cardiometabolic risk factors in adolescents: A 2-year longitudinal study

**DOI:** 10.3389/fspor.2022.1060530

**Published:** 2022-12-15

**Authors:** Karah J. Dring, Simon B. Cooper, Ryan A. Williams, John G. Morris, Caroline Sunderland, Gemma A. Foulds, A. Graham Pockley, Mary E. Nevill

**Affiliations:** ^1^ Sport Health and Performance Enhancement (SHAPE) Research Group, Department of Sport Science, School of Science and Technology, Nottingham Trent University, Nottingham, United Kingdom; ^2^ John van Geest Cancer Research Centre, School of Science and Technology, Nottingham Trent University, Nottingham, United Kingdom

**Keywords:** adolescents, cardiometabolic health, low-grade chronic inflammation, adiposity, physical fitness

## Abstract

Although risk factors for cardiometabolic diseases begin to present in young people, the association between physical fitness and adiposity with traditional and novel risk factors for cardiometabolic diseases across adolescence remains relatively unknown. Following ethical approval, fifty-two adolescents (age 11.6 ± 0.6 years; 32 girls) were recruited for a 2-years longitudinal study. Adiposity was assessed based on sum of skinfolds, waist circumference and body mass index, and physical fitness as distance run on the multi-stage fitness test (MSFT). Risk factors for cardiometabolic diseases (pro- and anti-inflammatory cytokines, plasma insulin, Homeostatic Model Assessment of Insulin Resistance - HOMA-IR, blood pressure) were measured following an overnight fast. Relationships between independent and response variables were analysed using multi-level modelling (final combined models were created using the stepwise backward elimination method). Plasma insulin concentration and HOMA-IR were positively associated with adiposity and inversely associated with distance run on the MSFT (all *p *< 0.05). The final combined models for plasma insulin concentration and HOMA-IR contained main effects for age, skinfolds and distance run on the MSFT. Levels of the anti-inflammatory cytokine IL-10 were inversely related to the sum of skinfolds (*p *= 0.046), whereas there was a trend for levels of the pro-inflammatory cytokine TNF-α to be positively related to the sum of skinfolds (*p *= 0.056). Adiposity and physical fitness are important, independent, determinants of metabolic health in adolescents. Furthermore, adiposity influences levels of pro- and anti-inflammatory cytokines in adolescence, with greater adiposity associated with a poorer inflammatory profile. The present study demonstrates an independent effect of physical fitness on metabolic health longitudinally across adolescence. It is therefore recommended that future work develops therapeutic interventions that reduce adiposity and enhance physical fitness in adolescents, to promote lifelong health.

## Introduction

The physical activity levels of children and adolescents Worldwide are below the recommended guidelines set by government and health officials, with up to 80% of boys and girls performing less than the recommended minimum of 60 min of moderate-to-vigorous physical activity per day (1). Longitudinal studies report that low physical activity levels in children and adolescents result in poorer physical fitness and increases in adiposity (2), which in turn are associated with increases in traditional risk factors for cardiometabolic diseases, such as blood pressure (3–5) and dyslipidaemia (3, 5).

Cross-sectional studies report that lower physical fitness and increased adiposity are associated with increased composite cardiovascular disease risk factor scores (3, 5). However, cross-sectional studies cannot infer causality in the physical fitness, adiposity and cardiometabolic health relationship. Consequently, longitudinal studies that prospectively examine young people are required to establish a causal effect of adiposity and physical fitness on cardiometabolic health (6–8). A recent meta-analysis (9) concluded that enhanced childhood cardiorespiratory fitness was associated with lower adiposity and reduced incidence of metabolic syndrome during adolescence. Whilst such findings are important, it is also necessary to determine whether the strength of the association between physical fitness, adiposity and cardiometabolic health changes during adolescence and whether a change in either adiposity or physical fitness affects the presence of risk factors for cardiometabolic diseases in this age group. Recently, a four-year longitudinal assessment of the physical fitness-cardiometabolic health relationship reported that improvements in physical fitness (multi-stage fitness test performance) were associated with reduced LDL cholesterol in boys (aged 8 years at baseline and 12 years at follow-up) and triglyceride concentration in boys and girls (6). In contrast, during a three-year follow-up, physical fitness (predicted maximal oxygen consumption from a progressive endurance test), when adjusted for baseline somatic maturity and cardiometabolic risk factors, was not associated with a cluster of traditional risk factors for cardiometabolic diseases (blood pressure, waist circumference, HDL cholesterol, triglyceride and blood glucose concentration) across adolescence (8). The discrepancies across the limited longitudinal studies to date potentially relate to the assessment of physical fitness (4), variations in the length of follow-up (6, 8), and differences in adjustment for confounding variables such as age, sex, and maturation (6–8). Furthermore, these studies have only considered traditional risk factors for cardiometabolic disease, such as blood pressure and blood lipid profile (6, 8).

It has recently emerged that novel risk factors for cardiometabolic diseases, such as the pro-inflammatory cytokines (IL-1β, IL-6 and TNF-α) that are implicated in the development of low-grade chronic inflammation, are inversely associated with physical fitness (assessed by performance on the multi-stage fitness test) in young people (4). Furthermore, levels of the anti-inflammatory cytokine IL-10, which is suggested to have a protective effect on cardiometabolic health (10), has also been reported to be positively associated with physical fitness (performance on the multi-stage fitness test) (4). These findings have clinical importance given the novel risk factors (levels of inflammatory cytokines and the acute phase protein C-Reactive Protein, CRP) for cardiometabolic diseases are deemed the best predictors of cardiovascular health in adults (11). However, the only evidence regarding the relationship between physical fitness and pro- and anti-inflammatory cytokines in young people is cross-sectional; with the limited longitudinal studies to date (6, 8) not measuring these novel risk factors. Furthermore, studies to date have not considered the effects of adiposity on these novel risk factors for cardiometabolic diseases across adolescence; nor has research to date considered whether the impact of such modifiable risk factors (adiposity and physical fitness) on low-grade chronic inflammation changes across adolescence.

Therefore, the aim of the present study was to determine longitudinally how the relationship between predictor variables (adiposity, physical fitness, and age) and traditional and novel risk factors for cardiometabolic diseases changes across adolescence. It was hypothesised, based on the available cross-sectional evidence to date, that the effect of physical fitness and adiposity on adolescent cardiometabolic health would strengthen through adolescence; with those with higher adiposity and lower physical fitness presenting with a more adverse cardiometabolic risk profile, with these effects being more pronounced later in adolescence following greater exposure to these risk factors.

## Materials and methods

### Participant characteristics

Fifty-two adolescents (aged 11.6 ± 0.6 years at baseline, 32 girls and 20 boys) volunteered to take part in this longitudinal study which comprised a baseline measurement (cross-sectional results published previously) (4) and an *a priori* follow-up measurement (2.2 ± 0.2 years later; range: 1.4–2.7 years). At baseline, 121 participants completed the study (4), of which 52 (43%) agreed to participate in the follow-up measurements reported in the present study. Participant characteristics at baseline and follow-up are presented in Table 1.

**Table 1:** Participant characteristics at baseline (age ∼11.6 years) and follow-up (age ∼13.8 years) for Male and female adolescents.

	Overall (*n* = 52)		Boys (*n* = 20)		Girls (*n* = 32)	
	Baseline	Follow-up	Baseline	Follow-up	Baseline	Follow-up
*Participant Characteristics*						
Age [y]	11.6 ± 0.6	13.8 ± 0.5^a^	11.8 ± 0.6	13.9 ± 0.5^a^	11.5 ± 0.6	13.7 ± 0.5^a^
Height [cm]	152.2 ± 7.4	165.7 ± 8.5^a^	155.7 ± 6.9	171.8 ± 8.1^a^	150.1 ± 6.9	161.9 ± 6.4^a^
Body mass [kg]	43.1 ± 9.1	53.6 ± 9.9^a^	43.5 ± 7.5	55.2 ± 9.1^a^	42.8 ± 10.1	52.6 ± 10.3^a^
BMI [kg^.^m^2^]	18.5 ± 3.3	19.5 ± 3.2^a^	17.9 ± 2.6	18.6 ± 2.3^a^	18.9 ± 3.7	20.1 ± 3.6^a^
Age from peak height velocity [y]	−1.9 ± 0.6	+0.1 ± 0.7^a^	−1.7 ± 0.8	0.4 ± 0.7^a^	−2.0 ± 0.5	−0.1 ± 0.6^a^
Sum of skinfolds [mm]	49.9 ± 27.0	47.4 ± 24.6	41.6 ± 17.8	34.5 ± 18.3^a^	55.1 ± 30.5	55.4 ± 24.8
Waist circumference [cm]	64.1 ± 7.0	67.0 ± 6.8^a^	65.1 ± 5.5	68.6 ± 4.6^a^	63.5 ± 7.8	66.0 ± 7.8^a^
Distance run on MSFT [m]	1,100 ± 500	1,320 ± 660^a^	1,460 ± 380	1,820 ± 480^a^	900 ± 460	1,040 ± 580^a^
*Response Variables*						
Plasma insulin concentration [pmol^.^L^−1^]	6.8 ± 4.7	12.9 ± 12.5	5.0 ± 2.4	8.3 ± 8.9	8.0 ± 5.5	15.9 ± 13.8
HOMA-IR	1.4 ± 1.1	2.5 ± 2.3	1.0 ± 0.6	1.8 ± 2.0	1.6 ± 1.2	3.0 ± 2.4
IL-6 [pg^.^ml^−1^]	3.2 ± 1.5	5.1 ± 1.7	3.2 ± 1.4	5.4 ± 2.1	3.3 ± 1.6	4.8 ± 1.3
IL-1β [pg^.^ml^−1^]	4.2 ± 3.3	4.6 ± 3.2	4.6 ± 4.3	4.4 ± 3.0	3.9 ± 2.5	4.7 ± 3.4
IL-10 [pg^.^ml^−1^]	2.7 ± 2.3	3.3 ± 2.3	2.6 ± 2.2	3.3 ± 1.8	2.7 ± 2.4	3.3 ± 2.5
TNF-α [pg^.^ml^−1^]	1.9 ± 2.0	10.8 ± 11.1	2.6 ± 2.8	9.1 ± 6.7	1.5 ± 1.1	12.0 ± 13.3
CRP [mg^.^L^−1^]	0.4 ± 0.5	0.8 ± 0.5	0.4 ± 0.6	0.8 ± 0.5	0.4 ± 0.4	0.8 ± 0.6
Mean arterial blood pressure [mmHg]	84 ± 7	86 ± 8	84 ± 7	85 ± 11	84 ± 7	87 ± 6

BMI, body mass index; MSFT, multi-stage fitness test.

^a^ Significantly different from baseline (*p* < 0.05).

### Study design

Ethical approval was received from Nottingham Trent University's Ethical Advisory Committee (Reference: SPOR-400) and all data were collected in accordance with the relevant guidelines and regulations that were approved by said ethical committee. The following exclusion criteria was applied during participant recruitment (a) a medical condition or medical history including but not limited to cardiovascular disease, diabetes, hypertension, (b) having an otherwise healthy family member that has died during or soon after exercise, (c) prescription of medication that would affect participation in the study, and (d) any factor that would affect the participants ability to complete the exercise components of the study. At baseline, participants were contacted *via* their school or sports club, located within the East Midlands, UK. At follow-up, contact was made *via* a telephone call to the participants' parent/guardian. Written parental consent and verbal assent from the participant was obtained at both baseline and follow-up. Health screen questionnaires were completed at baseline, and repeated at follow-up, by the participants' parent/guardian. These were subsequently checked by a Lead Investigator (KD, SC) to ensure there were no medical conditions that might affect participation in the study.

Each participant completed two assessments at baseline and follow-up, separated by a minimum of 7 days. Assessment one consisted of anthropometric measurements (height, body mass, waist circumference, skinfolds) and the multi-stage fitness test (MSFT), in that order. Assessment two consisted of rested and fasted blood pressure measures and capillary blood samples, in that order. Prior to each assessment, participants were asked to refrain from moderate-to-vigorous physical activity for 24 h. A telephone call was made to parents/guardians the evening prior to the assessment sessions to ensure compliance with the study requirements. Detailed descriptions of each of the assessments employed is provided elsewhere (4), as consequence of which only a brief description of each measurement is provided below.

### Anthropometric measurements

Participants underwent anthropometric measures of body mass and stature to calculate body mass index (BMI; calculated as body mass [kg]/stature [m]^2^) (4). Specifically, body mass was measured using a Seca 770 digital scale, accurate to 0.1 kg (Seca, Hamburg, Germany) and stature was measured using a Leicester Height Stadiometer, accurate to 0.1 cm (Seca, Hamburg, Germany), in line with our previous research (4).

### Body composition

In line with previous work (4), the sum of four skinfolds and waist circumference were used to measure adiposity. Skinfold thickness was measured using Harpenden Calipers (Baty International, Burgess, Hill, United Kingdom) at four sites (tricep, subscapular, supraspinale, front thigh). As per baseline measurements, skinfold thickness was taken twice in rotation and on the right-hand side of the body. An average of the two measurements was taken unless different by >5%, in which case a third measurement was taken, and the median value used as the criterion measure. All skinfold measures were completed by trained kinanthropometrists. Waist circumference was measured twice with a tape measure at the narrowest point of the torso between the xiphoid process and the iliac crest, to the nearest 0.1 cm. As per skinfold measurements, an average of the two waist circumference measurements was recorded unless different by >5%, in which case a third measurement was made and the median value used as the criterion measure.

### Multi-stage fitness test (MSFT)

All participants completed the MSFT, which consisted of progressive 20 m shuttle runs to the point of volitional exhaustion (12), in groups of 5–10. In brief, the MSFT commenced at 8.5 km.h^−1^ increasing in speed by 0.5 km.h^−1^ for each stage completed, in line with our previous research (4). Prior to the test, participants were fitted with a heart rate monitor (First Beat Technologies Ltd., Finland). Participants were paced by a member of the research team familiar with the MSFT and verbally encouraged throughout to ensure the test was completed to the point of volitional exhaustion (4). Total distance run was used as the criterion measure.

### Blood pressure

Following an overnight fast (from 9 pm the previous evening), participants arrived at the laboratory and were seated quietly for 5 min. Two blood pressure measurements were taken from the left arm, which was rested at chest height, using an HBP-1,300-United Kingdom sphygmomanometer (Omron, Milton Keynes, United Kingdom). The average of the two measures was used as the criterion value, unless systolic blood pressure differed by >5 mmHg, in which case a third measurement was taken, and the median used as the criterion measure. Mean arterial blood pressure was determined using the following calculation: diastolic blood pressure + {[0.33 ∗ (systolic blood pressure – diastolic blood pressure)]} (13).

### Capillary blood samples

A rested, fasted capillary blood sample was taken following the assessment of blood pressure. For this, participants’ hands were warmed *via* submersion in warm water to increase capillary blood flow and a Unistik single-use lancet (Unistik Extra, 21G gauge, 2.0 mm depth, Owen Mumford, Ltd., United Kingdom) was used to subsequently collect the blood into three 300 µl EDTA microvettes (Sarstedt Ltd., United Kingdom) (4). Blood samples were then centrifuged at 10,000 × * **g* for 4 min (Eppendorf 5,415C, Hamburg, Germany). Plasma was pipetted from the whole blood samples and distributed into three 500 µl plastic vials. All samples were frozen immediately at −20 °C and transferred to a −80 °C freezer as soon as possible. The methods described here are in line with our previous research (4).

Plasma insulin concentrations were determined using a commercially available ELISA (Mercodia Ltd., Sweden) (coefficient of variation = 3.2%). The homeostatic model assessment of insulin resistance (HOMA-IR) was calculated from fasted blood glucose and plasma insulin concentration (fasted plasma insulin (µU^.^mL^−1^) × fasted blood glucose (mmol^.^L^−1^)/22.5) (14). Pro-inflammatory (IL-1β, IL-6, TNF-α) and anti-inflammatory (IL-10) cytokine concentrations were determined using an AimPlex™ flow cytometry-based multiplex bead immunoassay kit (YSL Bioprocess Development Company, Pomona, United States) and analysed on a Gallios™ flow cytometer using Kaluza™ acquisition and analysis software (Beckman Coulter, London, United Kingdom). The intra-assay coefficient of variation based on 10 repeat measurements for pro- and anti-inflammatory cytokines using the methods described was 10.4%–15.9%.

### Statistical analysis

All data analyses were performed in the open source software programme R (www.r-project.org; version 3.5.1). The first step of the analyses was to check for collinearity between the independent variables, which was performed using a Pearson correlation. Collinearity was defined as *r* > 0.7. Subsequently, multi-level modelling was used to examine the relationship between the independent and response variables. All models were conducted using the *lme* function, which yields “*t*” statistics and is found in the *nlme* package in *R*. All models included age (normalised to age 11 years, i.e., the youngest participant in the data set) as a fixed effect. Random effects were included for the testing time point (i.e., baseline or follow-up) and for each participant. Prior to any analyses, the distribution of the response variable was inspected for normality; normal distributions were found for IL-6 concentration and mean arterial pressure. The remaining response variables exhibited non-normal distributions; these were subsequently log transformed prior to analysis to eliminate the right-hand skew in distribution (this applied to fasting plasma insulin concentration, HOMA-IR, IL-1β concentration, IL-10 concentration and TNF-α concentration).

Initially, individual models were run for each independent variable. Initial models included an interaction between age (as an indicator of time) and the independent variable in question. If this interaction was not significant, the interaction term was removed, and the main effects reported. For the development of the final models, any statistically significant independent variables that were not collinear (*r* < 0.7) were combined in the same model, and stepwise backwards elimination performed to arrive at the final model for each response variable. When statistically significant independent variables were collinear, a decision regarding which variable to include in the final model was based on the *t* value, *p* value and Akaike Information Criteria (AIC)/Bayesian Information Criteria (BIC). In multi-level modelling, the AIC and BIC are used as indicators of model fit, with smaller values indicating that the predictor variables in the model explain more of the variance in the outcome variable (when comparing models with the same number of observations). For all analyses, statistical significance was accepted as *p* < 0.05.

## Results

The cardiometabolic risk (response) variables at baseline and at follow-up 2 years later are shown in Table 1.

### Collinearity between predictor variables

Table 2 provides a correlation matrix for the independent variables (body mass, BMI, sum of skinfolds, waist circumference and distance run on the MSFT). To be considered for inclusion in the same model in subsequent analyses, a cut-off value of *r* < 0.7 was applied. The independent variables which had a correlation of *r* > 0.7 were considered collinear and not included in the same model.

**Table 2:** A correlation matrix to examine collinearity between the independent variables.

Body mass	BMI	Sum of skinfolds	Waist circumference	Distance run on MSFT
Body mass	*r* = 0.807^a^	*r* = 0.547	*r* = 0.869^a^	*r* = −0.166
	*p* < 0.001	*p* < 0.001	*p* < 0.001	*p* = 0.083
BMI		*r* = 0.862^a^	*r* = 0.899^a^	*r* = −0.501
		*p* < 0.001	*p* < 0.001	*p* < 0.001
Sum of skinfolds			*r* = 0.715^a^	*r* = −0.668
			*p* < 0.001	*p* < 0.001
Waist circumference				*r* = −0.296
				*p* = 0.002

^a^ *r* > 0.7 and thus these variables were considered collinear.

BMI, body mass index; MSFT, multi-stage fitness test.

### Multi-level models

Table 3 shows the results of the multi-level models conducted for each independent variable separately. Subsequently, the statistically significant (non-collinear) independent variables were combined to produce a model that best explained the variance in each response variable (Table 4). The following section presents the data for the effect of each independent variable (sex, body mass, BMI, sum of skinfolds, waist circumference, distance run on the MSFT) on the cardiometabolic risk (response) variables (plasma insulin concentration, HOMA-IR, IL-6, IL-1β, IL-10, TNF-α, mean arterial blood pressure), as assessed in the multi-level models.

**Table 3:** Results of the multilevel models considering the individual effects of each independent variable on each response variable.

Response Variable	Independent variable	Intercept	Age			Independent variable			Interaction			*Obs*	*AIC*	*BIC*	*Loglik*
			PE	SE	*p*	PE	SE	*p*	PE	SE	*p*				
Plasma Insulin concentration [pmol^.^L^−1^]^a^	Sex	2.576	0.149	0.068	0.035	−0.666	0.206	0.002			0.063	90	241.1	258.4	−113.6
	Body mass	0.201	−0.054	0.085	0.532	0.037	0.010	0.001			0.835	90	245.2	262.4	−115.6
	BMI	−0.516	0.066	0.068	0.334	0.121	0.027	<0.001			0.274	90	237.1	254.4	−111.6
	Sum of skinfolds	1.381	−0.191	0.129	0.147	0.005	0.005	0.356	0.007	0.002	0.004	90	240.3	259.9	−112.1
	Waist circumference	−1.176	0.056	0.072	0.437	0.045	0.014	0.002			0.822	90	246.2	263.4	−116.1
	MSFT distance	2.553	0.219	0.066	0.002	−0.017	0.003	<0.001			0.053	90	230.2	247.5	−108.1
HOMA-IR^a^	Sex	0.842	0.172	0.064	0.011	−0.613	0.206	0.005			0.254	90	235.5	252.8	−110.8
	Body mass	−1.301	−0.008	0.082	0.927	0.033	0.010	0.003			0.388	90	240.5	257.7	−113.2
	BMI	−2.085	0.096	0.064	0.146	0.116	0.026	<0.001			0.803	90	231.3	248.6	−108.7
	Sum of skinfolds	−0.461	−0.049	0.128	0.706	0.009	0.005	0.093	0.005	0.002	0.049	90	238.5	258.1	−111.2
	Waist circumference	−2.694	0.085	0.068	0.221	0.043	0.013	0.002			0.717	90	240.0	257.2	−113.0
	MSFT distance	0.846	0.241	0.063	<0.001	−0.016	0.003	<0.001			0.435	90	225.6	242.8	−105.8
IL−10 [pmol^.^L^−1^]^a^	Sum of skinfolds	0.916	0.132	0.048	0.009	−0.006	0.003	0.046			0.965	94	209.7	227.3	−97.8
	Waist circumference	1.823	0.166	0.050	0.002	−0.019	0.011	0.079			0.326	94	208.1	225.6	−97.0
TNF-α [pmol^.^L^−1^]^a^	BMI	1.367	0.565	0.091	<0.001	−0.069	0.040	0.092			0.195	86	282.2	299.1	−134.1
	Sum of Skinfolds	0.602	0.524	0.089	<0.001	−0.010	0.005	0.056			0.419	86	285.4	302.4	−135.7
	MSFT Distance	−0.323	0.500	0.091	<0.001	0.008	0.005	0.086			0.878	86	286.3	303.2	−136.2
Mean Arterial Pressure [mmHg]	Body mass	76.452	0.315	0.597	0.601	0.170	0.092	0.071			0.517	99	668.7	686.6	−327.3
	BMI	74.169	0.875	0.434	0.050	0.501	0.265	0.065			0.205	99	666.4	684.3	−326.2

PE, parameter estimate; SE, standard error; Obs, number of observations; AIC, akaike information criterion; BIC, bayesian information criterion; Loglik, log likelihood; BMI, body mass index; MSFT, multi-stage fitness test.

^a^ Indicates that the response variable is log transformed.

All independent variable PE are for a 1 unit change (e.g. 1 mm change in sum of skinfolds), except MSFT distance which is presented per 20 m shuttle.

The baseline for age is 11 years, the baseline for sex is girls; all other variables have the baseline of 0.

Example calculation for a main effect: *plasma insulin concentration for a 13 year old with a body mass of 45 kg =.*

Plasma insulin concentration [pmol^.^L^−1^]:

= exp(intercept + (age * age PE) + (body mass * body mass PE)).

= exp(0.201 + (2*−0.054) + (45*0.037)).

= exp(1.758) = 5.80 pmol^.^L^−1.^

Example calculation where an interaction is included: HOMA-IR for a 12 year old with a sum of skinfolds of 50 mm =.

Plasma insulin concentration [pmol^.^L^−1^]:

= exp(intercept + (age * age PE) + (sum of skinfolds * sum of skinfolds PE) + (age * sum of skinfolds * interaction PE)).

= exp(−0.461 + (1*−0.049) + (50*0.009) + (1*50*0.005)).

= exp(0.190) = 1.21.

**Table 4:** Results of the multi-level models showing the combination of independent variables that best explained the variance in each response variable.

Response Variable	Independent variables	Intercept	Model characteristics			*Obs*	*AIC*	*BIC*	*Loglik*
			PE	SE	*p*				
Plasma Insulin concentration [pmol^.^L^−1^]^a^	Age	2.197	−0.078	0.135	0.566	90	244.6	266.6	−113.3
	Skinfolds		−0.001	0.005	0.920				
	Age^a^ Skinfolds		0.006	0.002	0.021				
	MSFT Distance		−0.010	0.004	0.010				
HOMA-IR^a^	Age	0.167	0.223	0.063	0.001	90	238.9	258.5	−111.4
	Skinfolds		0.008	0.004	0.052				
	MSFT Distance		−0.0006	0.0001	0.004				

PE, parameter estimate; SE, standard error; Obs, number of observations; AIC, akaike information criterion; BIC, bayesian information criterion; Loglik, log likelihood; MSFT, multi-stage fitness test.

^a^ Indicates that the response variable is log transformed.

The baseline for age is 11 years; all other variables have the baseline of 0.

All independent variable PE are for a 1 unit change (e.g. 1 mm change in sum of skinfolds), except MSFT distance which is presented per 20 m shuttle.

Example calculation of plasma insulin concentration for a 14 year old with a sum of skinfolds of 50 mm and distance run of MSFT of 60 shuttles*.*

Plasma insulin concentration [pmol^.^L^−1^]:

= exp(intercept + (age*age PE) + (sum of skinfolds*sum of skinfolds PE) + (age*sum of skinfolds*interaction PE) + (MSFT distance*MSFT distance PE)).

= exp(2.197 + (3*−0.078) + (50*−0.001) + (3*50*0.006) + (60*−0.010)).

= exp(2.213) = 9.14 pmol.L−1.

#### Fasting plasma insulin concentration

The multi-level models (Table 3) demonstrated that all independent variables had a significant impact on fasting plasma insulin concentration across adolescence. Specifically, fasting plasma insulin concentration was lower in boys than girls (*p* = 0.002), increased as a result of increases in all measures of adiposity (body mass: *p* = 0.001; BMI: *p* < 0.001; waist circumference: *p* = 0.002), and decreased as distance run on the MSFT increased (*p* < 0.001).

**Figure 1:**
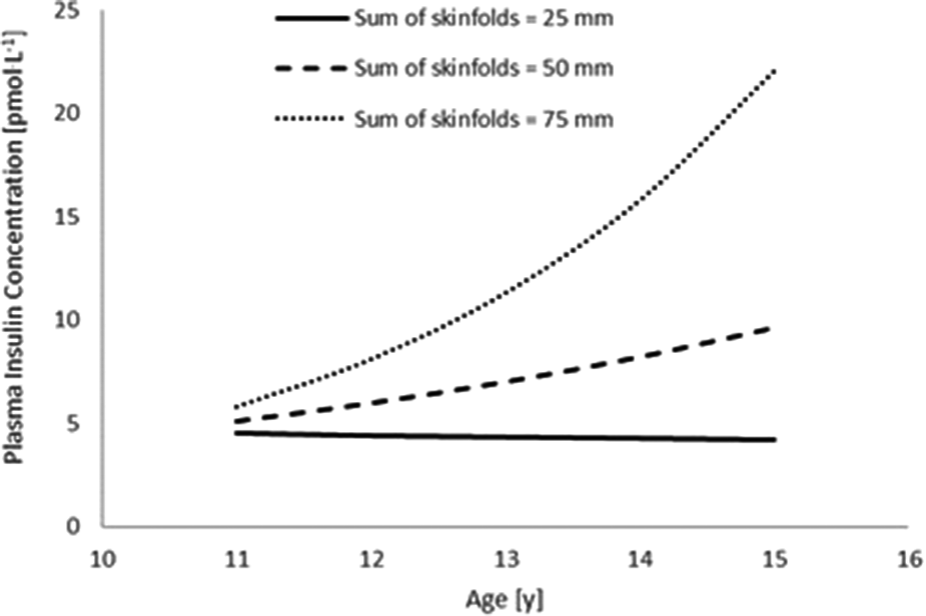
The interaction between sum of skinfolds and age, to affect plasma insulin concentration (*n* = 45). Modelled data display the mean sum of skinfolds (50 mm) ± 1 SD (25 mm and 75 mm) (age * sum of skinfolds interaction, *p* = 0.004).

Furthermore, sum of skinfolds also demonstrated an interaction with age (Table 3, Figure 1), whereby plasma insulin concentration decreased with age (main effect of age, *p* = 0.147), increased with an increase in sum of skinfolds (main effect of sum of skinfolds, *p* = 0.356), and also increased more per year for each unit increase in sum of skinfolds (age*sum of skinfolds interaction, *p* = 0.004).

**Figure 2:**
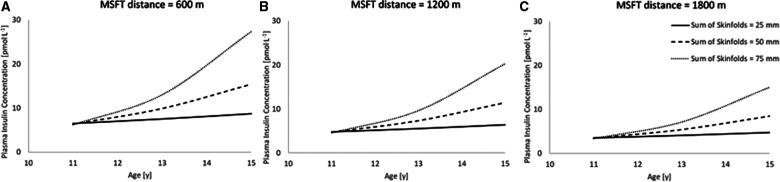
The combined model to best explain fasting plasma insulin concentration (*n* = 45). Modelled data for both distance run on MSFT and sum of skinfolds, where distance run on MSFT is 600 m (Figure 2A), 1,200 m (Figure 2B) and 1,800 m (Figure 2C). (Age * sum of skinfolds interaction, *p* = 0.021; main effect of distance run on the MSFT, *p* = 0.010). Hypothetical data displayed are mean ± 1 SD for both sum of skinfolds and MSFT.

Following the stepwise backwards elimination method, non-collinear independent variables were combined into the same model. The final model for fasting plasma insulin concentration contained an interaction between age and sum of skinfolds, in addition to a main effect for distance run on the multi-stage fitness test (Table 4; Figure 2), suggesting that these variables combine to best explain the variance in fasting plasma insulin concentration.

#### HOMA-IR

The multi-level models (Table 3) demonstrated that all independent variables had a significant effect on HOMA-IR. Specifically, HOMA-IR was lower in boys than girls (*p* = 0.005), increased as a result of increases in all measures of adiposity (body mass: *p* = 0.003; BMI: *p* < 0.001; waist circumference: *p* = 0.002); and decreased as distance run on the MSFT increased (*p* < 0.001).

**Figure 3:**
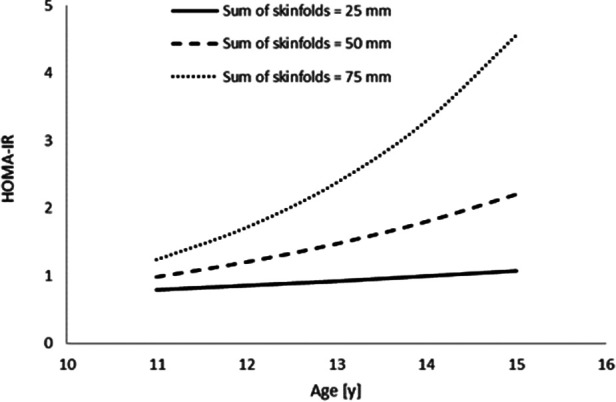
The interaction between sum of skinfolds and age, to affect HOMA-IR (*n* = 45). Modelled data display the mean sum of skinfolds (50 mm) ± 1 SD (25 mm and 75 mm) (age * sum of skinfolds interaction, *p* = 0.049).

Furthermore, sum of skinfolds also demonstrated an interaction with age (Table 3; Figure 3), in that HOMA-IR decreased with age (main effect of age, *p* = 0.706), increased as a result of an increase in sum of skinfolds (main effect of sum of skinfolds, *p* = 0.093), and also increased by more per year for each unit increase in sum of skinfolds (age*sum of skinfolds interaction, *p* = 0.049).

**Figure 4:**
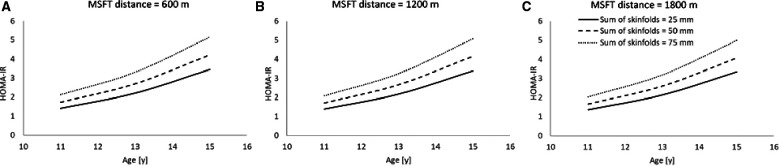
The combined model to best explain HOMA-IR (*n* = 45). Modelled data for both distance run on MSFT and sum of skinfolds, where distance run on MSFT is 600 m (Figure 4A), 1,200 m (Figure 4B) and 1,800 m (Figure 4C). (Main effect of sum of skinfolds, *p* = 0.052; main effect of distance run on the MSFT, *p* = 0.004). Hypothetical data displayed are mean ± 1 SD for both sum of skinfolds and MSFT.

Following the stepwise backwards elimination method, non-collinear independent variables were combined into the same model. The final model for HOMA-IR contained main effects for age, skinfolds and distance run on the MSFT (Table 4; Figure 4), suggesting that these variables combine to best explain the variance in fasting plasma insulin concentration.

#### Cytokines

The multi-level models showed there were no differences in fasting IL-6, IL-1β and CRP concentrations between boys and girls. In addition, the interindividual variations in cytokine levels could not be explained by any of the independent variables examined, or the interactions between the independent variables and age (Table 3).

However, the multi-level models (Table 3) demonstrated that sum of skinfolds and waist circumference influenced IL-10 concentration. Specifically, fasting IL-10 concentration decreased as the sum of skinfolds increased (*p* = 0.046) and tended to decrease as waist circumference increased (*p* = 0.079). However, IL-10 concentration was not affected by any other independent variable (all *p* > 0.05), nor were there any interactions between the independent variables and age (all *p* > 0.05). Following the stepwise backwards elimination method, non-collinear independent variables were combined into the same model. However, no combination of independent variables explained the variance in IL-10 concentration in the adolescents.

The multi-level models (Table 3) also demonstrated that BMI and sum of skinfolds influenced fasting TNF-α concentration. Specifically, fasting TNF-α concentration tended to increase as BMI (*p* = 0.092) and sum of skinfolds (*p* = 0.056) increased. Furthermore, TNF-α concentration tended to decrease as distance run on the MSFT decreased (*p* = 0.086). However, TNF-α concentration was not affected by any other independent variables (all *p* > 0.05), nor were there any interactions between the independent variables and age (all *p* > 0.05). Following the stepwise backwards elimination method, non-collinear independent variables were combined into the same model. However, no combination of independent variables explained the variance in TNF-α concentration in the adolescents.

#### Mean arterial blood pressure

The multi-level models (Table 3) demonstrated that body mass and BMI were the only independent variables which tended to affect mean arterial pressure (i.e., there was no effect of sex, sum of skinfolds, waist circumference or distance run on the MSFT). However, mean arterial pressure increased as body mass (*p* = 0.071) and BMI (*p* = 0.065) increased. There was no interaction between any of the independent variables and age (all *p* > 0.05, Table 3).

Finally, following the stepwise backwards elimination method, non-collinear independent variables were combined into the same model. However, no combination of independent variables explained the variance in mean arterial pressure in the adolescents.

## Discussion

The principal findings of the present study were that measures of adiposity (including BMI, sum of skinfolds and waist circumference) and physical fitness (measured by performance on the multi-stage fitness test) were both important, and additive, predictors of novel risk factors for cardiovascular disease (pro-inflammatory cytokine TNF-α and anti-inflammatory cytokine IL-10), blood pressure and metabolic health markers (plasma insulin concentration and HOMA-IR) across adolescence. Furthermore, the present study is the first, to the authors' knowledge, to track these risk factors across adolescence and to report that age, sex, adiposity, and physical fitness combine to explain the variance in insulin sensitivity (measured as fasted plasma insulin concentration and HOMA-IR) across adolescence.

The present study is the first to report that increased adiposity is associated with greater concentrations of pro-inflammatory cytokine TNF-α (BMI and sum of skinfolds) and lower concentrations of anti-inflammatory cytokine IL-10 (sum of skinfolds and waist circumference) in adolescents. These findings build upon previous research which only assessed adiposity *via* BMI and a clustered cardiometabolic health profile consisting of commonly cited cardiometabolic risk factors such as blood glucose concentration and blood pressure (8). Thus, the present study is original in that novel risk factors such as pro- and anti-inflammatory cytokine concentrations were measured, and multiple measures of adiposity were tracked across adolescence. The relationship between adiposity and levels of the pro-inflammatory cytokine TNF-α is particularly important, as it provides insight into our understanding of the mechanisms through which cardiometabolic diseases develop across the lifespan (15), given that TNF- *α* is implicated in the development of low-grade chronic inflammation (a risk factor for atherosclerosis) and insulin resistance (10). The inverse relationship between adiposity and levels of the anti-inflammatory cytokine IL-10 in children and adolescents identified in the present study corroborates previous evidence in adults, whereby weight loss (following restricted energy intake) in an overweight/obese population increased IL-10 concentration (16). As IL-10 is implicated in the inhibition of pro-inflammatory mediators IL-1β and TNF-α (15), it is hypothesised that through the maintenance of a healthy body composition, IL-10 concentration is increased and thus the development of low-grade chronic inflammation attenuated. Therefore, it is recommended that adiposity could be monitored to optimise cardiometabolic health and, for adolescents with increasing adiposity, therapeutic interventions could be designed to reduce adiposity and thereby attenuate the development of low-grade chronic inflammation.

Another novel finding of the present study was the interactions between age and adiposity (measured as sum of skinfolds) which influenced plasma insulin concentration and HOMA-IR across adolescence. Specifically, there was a greater increase in plasma insulin concentration and HOMA-IR with age in adolescents with higher adiposity (Figures 1, 3). Specifically, the findings suggest that those adolescents who are lower fit, and have a higher sum of skinfolds (i.e., greater adiposity), are likely to sit above the cut-off value for HOMA-IR (2.80) that indicates risk of metabolic syndrome in adolescents (17). This is particularly the case as the adolescents aged in the present study (Figures 3, 4). This increased presence of metabolic risk factors across adolescence is potentially explained by the increased exposure to adiposity across time. This is of concern given that adults who are diagnosed with type 2 diabetes as adolescents report more adverse symptoms (both microvascular and macrovascular complications) later in life than patients who were diagnosed with type 2 diabetes in adulthood (18). Therefore, the accumulating evidence for a role of exposure in disease severity in both adolescents (in the present study) and adults (18) emphasises the importance of maintaining a healthy body composition across the lifespan to prevent the early onset of metabolic risk factors, diseases such as type 2 diabetes and the resultant health complications.

Another key finding of the present study was that physical fitness (distance run on the MSFT) was inversely associated with metabolic risk factors (fasting plasma insulin concentration and HOMA-IR) and levels of the pro-inflammatory cytokine TNF-α, yet was not related to blood pressure or IL-6, IL-1β and IL-10 concentrations during adolescence. These findings build upon the findings of cross-sectional research which has shown lower physical fitness to be associated with increased composite cardiovascular disease risk scores (including blood pressure, waist circumference, triglyceride, LDL-c and blood glucose concentration) (3, 5) and levels of pro-inflammatory cytokines (IL-6 and IL-1β) in children and adolescents (4). However, the findings of the present study advance current understanding in the field, by reporting a longitudinal relationship between physical fitness and novel and traditional risk factors for cardiometabolic diseases across adolescence; and for the first time reporting the inverse relationship between physical fitness and TNF-α concentration in adolescents. Therefore, it is recommended that the MSFT be completed during adolescence as an indicator of cardiometabolic health. Furthermore, this suggests that therapeutic interventions should be designed to enhance the physical fitness of young people to prevent the development of cardiometabolic diseases; with encouraging evidence suggesting that physical activity interventions (particularly those that are high intensity) are an effective means of enhancing physical fitness in this population (19).

Finally, the present study reported novel insights into the combined effects of age, sex, adiposity, and physical fitness in predicting adolescent insulin sensitivity (measured as plasma insulin concentration and HOMA-IR). The combined models of the present study are the first to consider the longitudinal and combined effects of adiposity and physical fitness on risk factors for cardiometabolic disease across adolescence. The findings suggest that both adiposity (as assessed by sum of skinfolds) and physical fitness (as assessed by distance run on the MSFT), combine to explain more of the variance in plasma insulin concentration (Figure 2) and HOMA-IR (Figure 4), compared to the predictive ability of adiposity and physical fitness alone. In summary, an interaction existed between physical fitness and adiposity, in that young people with the poorest physical fitness and highest adiposity had significantly reduced insulin sensitivity when compared with counterparts who performed better on the MSFT or had lower adiposity, or a combination of the two (Figures 2, 4). Furthermore, the effect of low physical fitness and high adiposity on insulin sensitivity in adolescents increased with age (Figures 2, 4). As both sum of skinfolds and distance run on the MSFT provide low cost, relatively non-invasive, measures that can be implemented during adolescence to assess metabolic health, it is recommended that through regular measurement of adiposity and physical fitness, vulnerable young people could be identified for therapeutic intervention to prevent the development of metabolic risk factors and the early onset of type 2 diabetes.

The findings of the present study have important clinical significance, in that the factors affecting the presence of new risk factors for cardiometabolic diseases (increased TNF-α and reduced IL-10 concentration) have been identified in adolescents. However, when interpreting the findings of the present study the limitations of the research design should be considered, including the relatively short follow-up period of the participants (2 years), and the inclusion of only two measurement points (baseline and follow-up), which do not span adolescence in its entirety. A longer follow-up period across adolescence (tracking young people from aged 11 to 18 years), with more regular measurement intervals, is a challenging yet important avenue for future research. Furthermore, it must be considered that whilst 121 adolescents undertook baseline measurements (cross-sectional results reported elsewhere previously) (4), the present study reports on a follow-up of 52 adolescents. This is a relatively small sample size and caution should be taken when generalising the results to other populations. Furthermore, future research could also consider the effects of other variables that have been shown to impact health and well-being in young people, such as maturity status (20), diet (21), and sleep duration and efficiency (22). Additionally, future research could consider using technologies such as dual energy *x*-ray absorptiometry (DXA) for a more conclusive and detailed measurement of body composition. Such future studies would allow further insight to be developed regarding the relationship between adiposity, physical fitness, and age with risk factors for cardiometabolic diseases across adolescence, to ultimately improve the management of cardiometabolic health in young people.

## Conclusions

In conclusion, the findings of the present study provide important novel evidence that both adiposity and physical fitness are important, independent, determinants of metabolic health in adolescents. Furthermore, adiposity influences pro- and anti-inflammatory cytokine concentrations across adolescence, with greater adiposity resulting in a poorer inflammatory profile. Finally, the findings of the present study also demonstrate, for the first time, the additive benefits of low adiposity and high physical fitness for metabolic health in adolescents. Future work should develop therapeutic interventions that reduce adiposity and enhance physical fitness in young people, potentially through exercise and diet interventions, that can be adhered to long-term; with the aim of enhancing lifelong cardiometabolic health.

## Data Availability

The raw data supporting the conclusions of this article will be made available by the authors, without undue reservation.
